# Follow-up care for cancer survivors: the views of clinicians

**DOI:** 10.1038/sj.bjc.6605160

**Published:** 2009-07-28

**Authors:** D M Greenfield, K Absolom, C Eiser, S J Walters, G Michel, B W Hancock, J A Snowden, R E Coleman

**Affiliations:** 1 Academic Unit of Clinical Oncology, Weston Park Hospital, Sheffield, UK; 2 Department of Psychology, University of Sheffield, Sheffield, UK; 3 School of Health and Related Research, University of Sheffield, Sheffield, UK; 4 Department of Haematology, Royal Hallamshire Hospital, Sheffield Teaching Hospitals Foundation Trust, Sheffield, UK

**Keywords:** cancer survivors, follow-up care, specialist care, primary care

## Abstract

**Background:**

Evidence for the efficacy of late effects surveillance in adult cancer survivors is lacking and there is little agreement among clinicians on appropriate follow-up care.

**Methods:**

We report the views of both cancer experts and general practitioners (GPs) on long-term follow-up provision for cancer survivors, focussing on the 18–45 years age group. A total of 421 cancer experts (36% haematologists, 33% oncologists, 18% surgeons, 10% nurses, 2% other) and 54 GPs responded to a structured online survey. Reasons for follow-up care (clinical or supportive); advantages and disadvantages of follow-up in primary care; current practice; and resources required for a quality follow-up service were assessed.

**Results:**

Clinicians valued clinical reasons for follow-up more highly than supportive reasons (*P*<0.001). Learning more about late effects and checking for cancer recurrence were rated as the most important reasons for follow-up by cancer experts and GPs. A total of 85% of cancer specialists hold follow-up consultations alongside patients on active treatment. Cancer experts agreed that primary care follow-up would increase their availability for acute oncological care, but reduce information on late effects. The most important resource to provide a quality follow-up service was specialist nursing support (91%).

**Conclusions:**

Follow-up guidelines that include late effects surveillance are needed. Where and who should deliver this care requires further debate.

The prognosis of many cancers is steadily improving with over 50% of adult patients expected to survive for at least 5 years, regardless of age of onset (Cancer Research UK, 2008a). The most common malignancies in younger adults (aged 18–45 years) are breast cancer, germ cell tumours, lymphoma and leukaemia. Prognosis depends on tumour type, stage at diagnosis, and age at onset, with most cancers in younger adulthood having relatively good 5-year survival rates. For example, the 5-year survival probabilities for those aged 15–39 years (age range presented from data source) at diagnosis of breast or testicular cancers are 76 and 97%, respectively (Cancer Research UK, 2008b).

Despite the fact that many patients with neoplastic disease are successfully treated for their initial disease, cancer treatments may be associated with a variety of long-term side effects termed as ‘late effects’. These can be functional (for example, amputation, stomas), psychological (for example, anxiety, depression) or physical (for example second malignancy, thyroid disease, cardiac or respiratory dysfunction). Most of our understanding of late effects comes from the paediatric setting, in which it has been estimated that as many as 74% of long-term childhood survivors develop a chronic illness as a result of their original disease or treatment (Oeffinger *et al*, 2006). As a result, paediatricians have developed follow-up guidelines (Children's Oncology Group, 2004; Scottish Intercollegiate Guidelines Network, 2004; Skinner *et al*, 2005). In contrast, in the adult cancer setting, there are only a few national guidelines for clinicians on the identification or management of late effects. Exceptions include the Royal College of Physicians’ guidance on managing an early menopause in young women (Report of a Working Party November 2007 *et al*, 2008) and from a UK Expert Group, guidance for the management of breast cancer treatment-induced bone loss (Reid *et al*, 2008).

Recent documents, including the NICE Children's and Young People's Improving Outcomes Guidance (National Institute for Health and Clinical Excellence, 2005) and the National Cancer Survivorship Initiative born out of the Cancer Reform Strategy (Department of Health, 2007), both highlight the importance of long-term follow-up. Nevertheless, there is a paucity of evidence examining the efficacy of long-term follow-up in identification of late effects, particularly in younger adults who are likely to have more complex needs in terms of fertility, duration of survival, employment and family issues. Furthermore, the existing guidelines are essentially consensus rather than evidence-based. It is not clear, for example, what oncologists regard as clinically appropriate follow-up care. Anecdotal evidence suggests that clinical practice differs between cancer networks and is based on preferences of individual clinicians, even within the same tumour specialty.

The number of people in the United Kingdom living with and beyond cancer has been estimated as approximately two million, representing almost 4% of our population (Maddams *et al*, 2009). Currently, we have no information on the proportion of individuals who are disease-free, who are receiving regular follow-up or who have been discharged from the specialist centre. With the mounting burden of cancer survivors, it is likely that general practitioners (GPs) will take on more responsibility for routine follow-up for this population of patients, but it is unclear whether the primary care sector is either willing or able to take this on. However, in a rigorous trial of GP versus hospital follow-up of breast cancer, Grunfeld *et al*, 1996 reported that the majority of GPs wished to provide follow-up as long as certain provisos were assured. This study was limited only to breast cancer survivors and did not include late effects surveillance.

The aims of this study therefore were to determine and compare clinicians’ views of long-term follow-up care of younger adult survivors of common cancers (breast, germ cell, lymphoma and leukaemia) including reasons for follow-up care, advantages and disadvantages of follow-up in primary care, current practice, and the requirements for additional resources and supportive services.

## Materials and methods

An anonymous e-survey was targeted at cancer experts (oncologists, haematologists, surgeons and cancer nurses) who care for or specialise in breast cancer, lymphoma, leukaemia and germ cell malignancies, and at GPs (the questionnaire is available from authors on request). It was available online using an electronic web link in September and October 2007. Web links were sent by email through distribution lists and cascaded on. With this design, we are not able to estimate a response rate for this study. The initial invitation emails were endorsed and distributed by: the National Cancer Research Institute's Breast and Testis Clinical Studies Groups; the Royal College of Pathologists (list of haematologists); the British National Lymphoma Investigation; the National Cancer Network Lead Nurse Director (to expert cancer nurses); the Sheffield Primary Care Trust (to Sheffield General Practitioners). An initial invitation and two reminders were sent, except for haematologists who received only one reminder in an electronic newsletter from the Royal College of Pathologists.

In a preface to the survey, cancer survivors were defined as being disease-free and a minimum of 2 years from diagnosis. We also defined late effects as ‘physical and psychological problems that can occur months or years after the completion of cancer treatment.’ We particularly asked respondents to focus on the care and management of adults aged between 18–45 years, as younger adults have historically been the explicit focus of our previous research and clinical interest. Cancer experts were specifically asked to select their main tumour group (breast, lymphoma, leukaemia or germ cell) and respond to questions addressing the follow-up care of the selected tumour-specific cancer survivors. Given that the focus of the survey was service evaluation, ethics committee approval was not required, although we sought local approval for the GP survey to access Sheffield GPs through the Sheffield PCT's local medical committee.

The questionnaire was organised around the following sections:

1. Demographic information. Gender, profession, number of years of experience.
2. Reasons for attending follow-up care. Respondents rated the importance of follow-up on two scales measuring clinical care (five items; for example, cancer-related medical care) and supportive care (four items; for example, psychosocial, health behaviour advice) (adapted from (Absolom *et al*, 2006)). Items were rated on five-point scales (1–5) with higher scores indicating more importance. For analysis, the two categories important/very important have been combined, and this proportion has been reported along with raw number of responses and confidence intervals.
3. Advantages and disadvantages of follow-up in primary care. We listed six possible advantages (for example, least expensive option, allows cancer specialist to focus on acute care, easier for patients to ask GP's advice) and five possible disadvantages of follow-up in primary care (for example, too many calls on GP's time, loss of late-effects information, lack of expertise in primary care). Items were developed in consultation with local GPs, oncologists and haematologists. Items were rated on five-point scales (1–5) with higher scores indicating more agreement. For analysis, the categories agree/strongly agree have been combined, and this proportion has been reported with the raw number of responses and confidence intervals.
4. Current clinical practice. Respondents were asked whether they used a tumour-specific follow-up protocol. Cancer experts were additionally asked about their current clinical practice, such as organisation of follow-up care and use of protocols for discharging cancer survivors (yes/no responses).
5. Resources required to provide a quality follow-up service. We listed five possible resources needed to provide a meaningful follow-up service (for example, standardised guidelines; financial resources; specialist nursing support) (yes/no responses).
6. Open text boxes. They were available throughout for additional comments and alternative suggestions.

### Analysis

#### Sample size

One of the aims of the survey was to estimate clinicians’ views of long-term follow-up of cancer survivors. Assuming that a proportion of 50% would respond positively to a question on long-term follow-up, with 400 responders to the survey, we would be able to estimate this proportion within ±5%, that is, 95% confidence interval from 45 to 55% (Altman *et al*, 2000). The online survey was technically managed by NHS Healthcare Assessment and Training (HcAT) and anonymous data were stored on an electronic database. Data were transferred to the research team and analysed using SPSS v11 (SPSS Inc., Chicago, IL, USA) for Mac. Frequencies, means and ranges were calculated, and where appropriate, rank scores were generated. *χ*^2^, *t*-tests and one way analysis of variance (ANOVA) with a *post hoc* (Tukey's) test to allow for multiple comparisons, were used to compare differences of opinions between either (a) tumour group specialist (for example, between breast cancer and germ cell specialist) or (b) occupation groups (for example, between nurses and oncologists). A *P*-value of <0.05 was regarded as statistically significant. Ninety-five percent confidence intervals for the proportions responding to a statement were also calculated (Altman *et al*, 2000). Additional comments from open text boxes were examined and themed using content analysis by two independent coders and ratified by a third coder.

## Results

### Sample

A total of 421 cancer experts and 54 GPs responded to this online survey. Clinical and demographic details of respondents are shown in Table 1.

### Reasons for attending follow-up care

**Figure 1:**
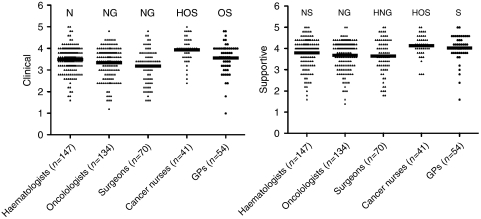
Scatter plots of scores for clinical and supportive reasons for follow-up by professional group. Scores were rated on a five-point (1–5) scale with a high score indicating more agreement. Mean scores were compared between professional groups by a one-way ANOVA (analysis of variance). If the overall one-way ANOVA was significant at the 0.05 level, then a series of pairwise multiple comparisons were made to determine which mean differences using a *post hoc* (Tukey's) test allowed for multiple comparisons. Significant differences between the mean scores by professional groups are indicated using the following notation: H=haematologist, O=oncologist, S=surgeon, N=nurse, G=GP. For example, for clinical care scores, the N above the haematologist scatter plot implies significant differences between the mean haematologist and nurse scores, but none of the other professional groups.

The mean scores for clinical and supportive reasons of follow-up were compared within each professional group (Table 2). Overall, clinicians rated clinical reasons for follow-up care more highly than supportive reasons (3.8 *vs* 3.5, difference 0.3, 95% CI: 0.3–0.4; *P*<0.001). One-way analysis of variance comparing mean scores between the professional groups also showed significant differences (Figure 1). Cancer nurses rated both clinical and supportive reasons higher than did all other cancer experts but similar to GPs. Surgeons rated supportive reasons significantly lower than did haematologists but similar to oncologists.

Cancer experts rated learning more about late effects (76%, 316 out of 421; CI 72–80%) as the most important reason for follow-up (Table 3). Checking for cancer recurrence was of secondary importance (71%, 295 out of 421; CI 66–75%), whereas 94% (51 out of 54; CI 85–98%) of GPs rated checking for cancer recurrence as the most important reason for follow-up, a difference of 23% (CI 13–29%, *P*<0.001).

Content analysis on open-ended responses confirmed this basic distinction between clinical and supportive reasons for follow-up and included additional benefits such as monitoring outcomes, research/clinical trial follow-up, improving staff morale, training and fund-raising. The drawbacks of follow-up were categorised as: patient related (false reassurance, perpetuates sick role, extra costs incurred by the patient, anxiety, results in delays in investigations or early detection); clinic related (expense, workload, lack of evidence of follow-up efficacy); role related (lack of continuity of care, responsibility falls mainly on inexperienced junior medical staff, the role of the GP is undermined and there is an inappropriate use of specialist time addressing general medical problems). Benefits from hospital-based follow-up were identified in terms of access to patient support groups and continuity of care in the specialist setting.

### Follow-up in primary care

#### Advantages

**Figure 2:**
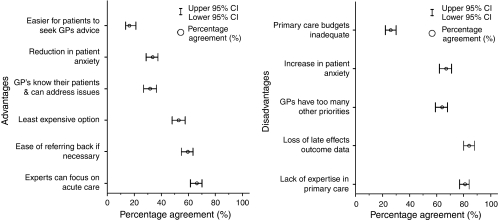
Advantages and disadvantages of follow-up in primary care according to cancer experts.

A total of 69% of cancer experts (276 out of 402; CI 64–73%) agreed that the most important advantage of follow-up in primary care is to enable cancer specialists to focus on acute care (Figure 2). Other high-scoring advantages of primary care follow-up included ease of referring back to the specialist centre as required (63%, 253 of 400; CI 58–68%) and the least expensive option (55%, 221 out of 399; CI 50–60%). From the GP perspective, their existing relationship with patients (82%, 40 out of 49; CI 69–90%), their accessibility (69%, 34 out of 49; CI 55–80%) and lower costs (66%, 33 out of 50; 52–78%) were considered as distinct advantages of follow-up in primary care. Statistically significant differences in mean scores between cancer experts and GPs were observed for GP accessibility (2.6 *vs* 3.6, difference −0.9, 95% CI: −1.3 to −0.7; *P*<0.001), GPs’ existing relationship with patients (2.9 *vs* 3.8, difference −0.9, 95% CI: −1.1 to −0.7; *P*<0.001) and reduction in patient anxiety (2.9 *vs* 3.4, difference −0.4, 95% CI: −0.7 to −0.2; *P*=0.001).

Content analysis of open-ended responses from cancer experts identified other advantages which were categorised as patient related (normalises the patient experience, encourages independence from specialist team, reduces patient travel and cost); GP skill related (more experience than cancer specialist in identifying and managing common medical problems, health promotion advice); systems related (knock on benefits in reducing cancer waiting times for new referrals by releasing more appointments, mechanisms for expediting direct referrals back to specialist care).

Content analysis of GPs’ open-ended responses identified benefits of follow-up in primary care as patient related (giving patients a choice) and role related. Several examples of role-related benefits were proposed. For example, GPs are well placed to offer supportive follow-up, including advice on health behaviours, reassurance about health and everyday advice. In addition, GPs may help to reduce the sense of abandonment that a patient may feel after discharge from a specialist centre.

#### Disadvantages of follow-up in primary care

A total of 84% (339 out of 402; CI 80–88%) of cancer experts rated the potential loss of outcome data and information on late effects as the main disadvantage of follow-up in primary care (Figure 2). Other key disadvantages rated by cancer experts were the lack of expertise in primary care (81%, 327 out of 405; CI 77–84%) and the potential increase in patient anxiety (69%, 280 out of 405; CI 64–73%). In comparison, 79% (41 out of 52; CI 66–88%) of GPs viewed inadequate budgets, too many other demands on their time (77%, 40 out of 52; CI 64–86%) and lack of appropriate expertise (75%, 39 out of 52; CI 62–85%) as the main potential disadvantages of follow-up in primary care. Statistically significant differences in the ranking of disadvantages between cancer experts and GPs were observed for inadequate primary care budgets (3.1 *vs* 3.8, difference −0.8, 95% CI: −1.1 to −0.6; *P*<0.001), too many other priorities for GPs (3.6 *vs* 3.9 difference −0.4, 95% CI: −0.6 to −0.1; *P*=0.005), loss of outcome data (4.1 *vs* 3.6, difference 0.4, 95% CI: 0.2–0.7; *P*=0.001) and lack of expertise (4.1 *vs* 3.8, difference 0.3, 95% CI: 0.1–0.5; *P*=0.018).

Content analysis on both cancer experts and GPs’ open-ended responses identified other disadvantages, including patient-related concerns (anxiety, choice, conveys notion of cure and false reassurance), specialist-related concerns (patient trust), GP organisation-related concerns (reticence, inadequate mechanisms of re-referral, already have too many protocols, inadequate resources) and GP skill-related concerns (delays in investigation and re-referral, GP training and expertise deficit, lack of mutual confidence between specialist and primary care).

### Current clinical practice

None of the GPs reported using a tumour-specific follow-up protocol (0%, 0 out of 55 CI 0–7%). This compared with 43% (176 out of 413; CI 38–47%) of cancer experts (76% (56 out of 74; CI 65–84%) surgeons, 58% (79 out of 137; CI 49–66%) oncologists, 51% (22 out of 43; CI 37–65%) nurses, 10% (15 out of 150; CI 6–16%) haematologists). Protocols were used by 62% (118 out of 190; CI 55–69%) of breast cancer experts, 84% (16 out of 19; CI 62–94%) of germ cell experts, 19% (26 out of 137; CI 13–26%) of lymphoma experts and 19% (7 out of 37 CI 9–34%) of leukaemia experts.

A total of 85% of cancer experts (331 out of 390; CI 81–88%) reported seeing follow-up patients alongside those receiving active treatment. This did not differ significantly by tumour group. Only 5% (14 out of 266) of cancer experts discharge patients at 2 years from end of treatment; 60% (196 out of 324) discharge patients by 5 years from end of treatment (80% (119 out of 149; CI 73–86%), 71% (12 out of 17; CI 47–87%), 42% (45 out of 108; CI 33–51%) and 32% (10 out of 31; CI 19–50%), respectively, for breast, germ cell, lymphoma and leukaemia experts).

### Resources required for a quality follow-up service for cancer survivors

A total of 91% of respondents (405 out of 447; CI 88–93%) rated specialist nursing support as the most important resource required for a quality follow-up service. This was followed by financial resources (84%, 372 out of 441 CI 81–87%) and standardised guidelines (79%, 354 out of 449 CI 75–82%). The responses did not differ significantly by professional or tumour group.

## Discussion

This survey specifically explored the views of clinicians from a broad range of professional groups and from across several tumour specialties towards follow-up services for younger adult cancer survivors aged 18–45 years. Provision of long-term follow-up care is topical and this survey coincides with national developments to redesign the clinical service for cancer survivors. We found that regardless of specialty, clinicians value clinical reasons for follow-up more highly than supportive reasons, with ‘learning more about late effects’ as the top priority. Differing expectations between patients and professionals may play a part, with cancer specialists not feeling that offering supportive care is part of their remit. Furthermore, cancer experts were particularly concerned that transfer of care to the GP would result in the loss of outcome data on late effects. The recent Department of Health recall for breast screening of young women treated for Hodgkin's lymphoma with mantle radiotherapy (Department of Health, 2003) is an example of why recording outcome data is important. All clinicians agreed that follow-up in primary care would enable cancer specialists to focus more on acute care. Notably, 91% of all clinicians indicated that the most important resource to provide a quality follow-up service is specialist nursing support.

Understandably, the delivery of acute care remains the number one priority in cancer management. Improvements in palliative and supportive care have been significant in recent years, whereas follow-up programmes, where they exist, have focused on detecting recurrence. Indeed, in our survey ‘checking for cancer recurrence’ was identified as the most important reason for follow-up by GPs and was also scored very highly by cancer experts. In the immediate period after the end of treatment, there may be a high risk of recurrence, with rates differing according to tumour group; hence, this finding is unsurprising. However, justifying follow-up mainly on the grounds of cancer recurrence surveillance may be a false reassurance for patients, as most cases are detected between scheduled follow-up appointments (Grunfeld *et al*, 1996). As time since the end of treatment increases, recurrence rates lessen. Follow-up will not reduce recurrence rates, although early identification in some situations may influence the outcome (Renehan *et al*, 2002) and the success or otherwise of salvage options, as some relapses are curable (for example, lymphoma or a loco-regional recurrence from breast cancer). Thus, research is required to investigate the clinical effectiveness of follow-up provision for cancer recurrence, as well as the screening for and management of late effects.

Cancer experts and GPs both agree that late effects expertise in primary care is currently insufficient and as such is currently a distinct disadvantage of follow-up in this setting. A number of advantages were identified, however, including GPs experience in managing chronic illness, familiarity with their patients, geographical proximity and convenience. Furthermore, GPs identified their own expertise as both provider of and referral to local supportive care agencies. On the other hand, with only a small number of cancer survivors registered at each practice, following a tumour-specific follow-up may not be feasible.

Whether the disadvantages of follow-up in primary care outweigh the potential advantages is unclear, and likely to vary depending on the underlying diagnosis and extent of earlier treatment. A risk-adapted strategy with patients perceived at low risk for recurrence and serious late effects might be best suited to follow-up in primary care, whereas those with more complex cases and those still at high risk of cancer recurrence may be better served by hospital-based follow-up. A shared-care approach, with the specialist centre prescribing appropriate medical surveillance through a treatment summary and long-term follow-up care plan, delivered by the GP, may prove an alternative model. The caveat with any proposed model of follow-up is patient acceptability. Results of a systematic review examining the evidence of alternative models of follow-up in breast cancer (Montgomery *et al*, 2007) indicated that different care options may be acceptable to patients and may also be associated with other benefits. Moreover, our survey of patients (Absolom *et al*) indicated that cancer survivors would prefer follow-up to remain in the specialist setting, although they might be willing to try alternative models.

In terms of current practice, 85% of cancer specialists surveyed held follow-up consultations with cancer survivors alongside patients on active treatment. Under these circumstances, most follow-up patients receive short appointments, often with junior medical staff, who, although capable, do not offer continuity. Beaver and Luker (2005) found that, although breast cancer survivors gained reassurance from a brief follow-up consultation focusing on detection of recurrent disease, there were few opportunities to meet their information and psychosocial needs. Indeed, our parallel survey of long-term survivors (Absolom *et al*, 2009) indicated that patients themselves view clinical reasons for follow-up more highly than supportive, indicating, perhaps, that they have low expectations of receiving supportive care within the current follow-up framework.

Results from our survey and those of others (Donnelly *et al*, 2007) identified that most breast cancer experts are not discharging patients at 3 years post diagnosis, as per the NICE 2002 guidelines (National Institute for Clinical Excellence, 2002). Particular concerns of experts rendering the guidelines obsolete include the need to provide adequate guidance on long-term endocrine treatment (where biologically appropriate), or on the risk-benefits of breast reconstruction and available surgical options, areas generally beyond the remit of follow-up in primary care and best addressed in a specialist setting. The new updated NICE breast guidelines (National Institute for Health and Clinical Excellence, 2009) have been amended to address follow-up issues with a particular recognition that follow-up care needs to be personalised.

Almost one-third of cancer experts who responded to this survey were haematologists and, of note, only 10% reported using a cancer-specific follow-up protocol. The long-term needs of, for example, the haemato-oncology patient after stem cell transplantation conditioned with total body irradiation, are extremely complex with almost any system and organ of the body at risk of late effects (Socié *et al*, 2003; Tichelli *et al*, 2008), and important sequelae may need to be addressed on a case by case basis. Although essentially consensus rather than evidence-based, long-term follow-up guidelines are used widely in a paediatric setting (Children's Oncology Group, 2004; UKCCSG Late Effects Group, 2005). These may provide a useful starting point to be adapted for use in an adult oncology setting.

Our survey identified financial resources, standardised guidelines and late effects expertise, as the most important resources required to provide a meaningful, quality follow-up service, with specialist nursing support identified as the critical resource for such a service. This is in concordance with the Cancer Reform Strategy (Department of Health, 2007). There is a small body of evidence indicating that nurse-led follow-up is clinically effective and acceptable to the patient (Moore *et al*, 2002; Cox and Wilson, 2003; Knowles *et al*, 2007; Beaver *et al*, 2009), but more research is needed in this area, particularly focussing on clinical effectiveness of late effects surveillance.

Our survey had limitations. We were unable to establish a response rate as the web link to the survey was distributed electronically by e-mail and cascaded on through a variety of mailings. Despite a sizeable sample, we were unable to ensure that our survey respondents are representatives of the entire target audience. In addition, the GPs in this survey are from one geographic region only, and attitudes may vary from one region to another, and between urban and rural practices.

Follow-up care is being increasingly recognised both nationally (Department of Health, 2007) and internationally as a pressing health issue (Grunfeld, 2006; Herbst *et al*, 2006). Long-term follow-up for all cancer survivors, regardless of age, may change with eventual recommendations from the National Cancer Survivorship Initiative. As a baseline, the survivorship initiative acknowledges that there are already a myriad of agencies offering supportive care, including both professional and voluntary services, and is examining ways in which integrated pathways between supportive care and clinical care can be developed. Rising numbers of cancer survivors mean that the burden of care needs addressing and that any changes require robust evaluation to examine both their clinical effectiveness and acceptability. The findings from our survey highlight some important priorities, needs and opinions amongst health care providers around which new services need to be shaped.

## Figures and Tables

**Table 1:** Characteristics of responders (*n*=475)

	**All**	**Haematologist**	**Oncologist**	**Surgeon**	**Cancer nurse**	**Other cancer experts**	**GP**
*Sex*							
Male, *n* (%)	274 (58)	90 (59)	82 (59)	65 (88)	5 (11)	3 (30)	29 (54)
Female, *n* (%)	191 (40)	61 (40)	58 (41)	6 (8)	38 (86)	7 (70)	21 (39)
Undeclared/missing, *n* (%)	10 (2)	2(1)	0 (0)	3(4)	1 (2)	0 (0)	4 (7)
Total, *n* (%)	475 (100)	153 (100)	140 (100)	74 (100)	44 (99)	10 (100)	54 (100)
							
*Years experience*							
Mean years, (*n*, s.d.)	19 (462, 8)	19 (151, 8)	18 (138, 7)	21 (71, 8)	15 (43, 7)	14 (10, 7)	21 (49, 9)
							
*Hospital type*							
District general, *n* (%)	222 (53)	91 (60)	55 (39)	44 (60)	24 (55)	8 (80)	
Metropolitan teaching, *n* (%)	181 (43)	56 (37)	79 (56)	28 (38)	18 (41)	0 (0)	
Undeclared/missing, *n* (%)	18 (4)	6 (4)	6(4)	2 (3)	2 (5)	2 (20)	
Total, *n* (%)	421 (100)	153 (101)	140 (99)	74 (101)	44 (101)	10 (100)	
							
*Tumour specialty*							
Breast cancer, *n* (%)	190 (45)	0 (0)	94 (67)	63 (85)	27 (61)	6 (60)	
Lymphoma, *n* (%)	140 (33)	102 (67)	19 (14)	8 (11)	8 (18)	3 (30)	
Leukaemia, *n* (%)	37 (9)	32 (21)	0 (0)	3 (4)	2 (5)	0 (0)	
Germ cell, *n* (%)	21 (5)	0 (0)	19 (14)	0 (0)	2 (5)	0 (0)	
Miscellaneous/missing, *n* (%)	33 (8)	19 (12)	8 (6)	0 (0)	5 (11)	1 (10)	
Total, *n* (%)	421 (100)	153 (100)	140 (101)	74 (100)	44 (100)	10 (100)	
							
*GP status*							
Practice partners, *n* (%)							44 (81)
Salaried, *n* (%)							8 (15)
Undeclared, *n* (%)							2 (4)
Total, *n* (%)							54 (100)

Abbreviation: GP=general practitioner. % rounded up or down to nearest integer.

**Table 2:** Comparisons of clinical and supportive reasons for follow-up by professional group

	**Reasons for follow-up**							
	**Clinical**		**Supportive**		**Mean**	**95% CI**		
**Professional group (*n*)**	**Mean**	**s.d.**	**Mean**	**s.d.**	**Difference**	**Lower**	**Upper**	***P*-value**
All clinicians (456)	3.8	0.8	3.5	0.7	−0.3	−0.4	−0.3	<0.001
Haematologists (147)	3.8	0.8	3.5	0.6	−0.3	−0.4	−0.2	<0.001
Oncologists (134)	3.7	0.7	3.3	0.7	−0.3	−0.4	−0.3	<0.001
Surgeons (70)	3.7	0.8	3.2	0.8	−0.5	−0.6	−0.4	<0.001
Cancer nurses (41)	4.1	0.5	3.9	0.6	−0.2	−0.3	−0.1	0.001
GPs (54)	4.0	0.6	3.6	0.7	−0.5	−0.6	−0.3	<0.001

Abbreviations: CI=confidence interval; GP=general practitioner.

Scores were rated on a five-point (1–5) scale with a high score indicating more agreement. Clinical care (for example, cancer-related medical care). Supportive care (for example, psychosocial, health behaviour advice). *P*-value from paired *t*-test.

**Table 3:** Reasons for attending follow-up: clinicians’ ratings

**Item**	**Scale**	**Rank (cancer experts)**	**% Cancer experts reporting important or very important**	**Rank (GPs)**	**% GPs reporting important or very important**
Learn more about late effects	C	1	75.8	=3	83.3
Check for cancer recurrence	C	2	70.8	1	94.4
Provide patients with information about potential late effects	C	3	70.1	=3	83.3
Provide patients with psychological support	S	4	69.6	5	79.6
Reassure patients about their health	C	5	69.4	2	87.0
Provide patients with best medical care	C	6	57.9	6	70.4
Give patients the opportunity to talk to staff who understand	S	7	52.8	8	59.2
Advise patients on how to keep healthy	S	8	41.6	7	64.8
Provide advise on everyday things such as insurance	S	9	27.0	9	38.9

Abbreviations: C=clinical; GP=general practitioner; S=supportive.
